# Comparison of treatment methods for pediatric Monteggia fracture: Met vs missed radial head dislocation

**DOI:** 10.1097/MD.0000000000013942

**Published:** 2019-01-11

**Authors:** Jin Peng He, Yun Hao, Jing Fan Shao

**Affiliations:** a Department of Pediatric Surgery; b Department of Radiology, Tongji Hospital, Tongji Medical College, Huazhong University of Science and Technology, Wuhan, Hubei 430030, China.

**Keywords:** children, missed dislocation, Monteggia fracture, treatment

## Abstract

Various methods are applied in the treatment of fresh and neglected Monteggia fractures. The purpose of this retrospective study was to evaluate the efficacy of various treatment methods, and assess the complexity associated with missed radial head dislocation.

All fracture patients were reviewed between Jan 2012 and Dec 2016. A detailed comparison was made of the treatment methods between fresh Monteggia fractures and neglected Monteggia fractures with missed diagnosis of dislocation.

A preliminary analysis of clinical information from 1081 patients in our center was investigated, and 42 were included in the final analysis. The fresh group included 25 patients with an average Mayo Elbow Performance Score of 96.3 ± 2.7 and resulted in the following scores after treatment: 21 excellent, 3 good, and 1 fair. In the fresh group, 76% of patients received closed reduction. Treatment with a cast, elastic stable intramedullary nail, and the Kirschner wire stabilization with tension band wiring make up 80% of the choices for fixation treatment. No patients experienced associated vascular injuries, recurrent dislocation, or elbow dysfunction. The neglected group involved 17 patients with Mayo Elbow Performance Score of 92.1 ± 9.3 and resulted in the following scores after treatment: 10 excellent, 4 good, and 3 fair. The locking compression plate (LCP) was the most common choice for postoperative immobilization in the neglected group (88.2%). Three patients in the neglected group experienced recurrent dislocation.

This retrospective analysis indicates that the treatment of neglected Monteggia fractures is more complex than that of fresh Monteggia fractures, and usually results in a worse recovery rate with a higher rate of recurrent dislocation and elbow dysfunction.

## 1 Introduction

An upper extremity injury involving a proximal ulna fracture with an associated radial head dislocation was first described by Italian surgeon Giovanni Monteggia.^[1]^ Until 1967, Monteggia fractures were further classified into four main types and 2 equivalent lesions by José Luis Bado.^[1]^ The Bado classification system, that is based on the direction of dislocation of the radial head, has been broadly used for guiding the treatment of Monteggia fractures. Bado type I describes the most common type of Monteggia fractures.

The key feature in the management of Monteggia fractures is to ensure the stability of the reduced radial head. A radial head dislocation that goes undiagnosed for longer than 4 weeks is considered neglected.^[2–4]^ Treatment of a neglected radial head dislocation in children remains a challenge for pediatric orthopedic surgeons. The primary issue with neglected radial head dislocation or subluxation is that it can lead to chronic elbow disability, progressive deformity, and loss of motion, particularly supination and pronation. This leads to a high probability of the requirement of an invasive operation and elbow dysfunction. Therefore, we carried out a comparison of the treatment methods employed for fresh Monteggia fractures and neglected Monteggia lesions, in order to improve understanding of the diagnosis and treatment of forearm fractures.

## 2 Materials and methods

Between Jan 2012 and Dec 2016, 1081 pediatric patients underwent surgery for bone fracture in our trauma center (Department of Pediatric Surgery, Pediatric Orthopedics Sub-specialty, Tongji Hospital, Tongji Medical College, Huazhong University of Science and Technology). Overall, 190 pediatric patients underwent surgery for forearm fracture, and only 42 patients underwent surgery for Monteggia fracture. Any Monteggia like-lesions were not included in this study. Therefore, a total of 42 patients were included in this retrospective study.

The cohort included 10 girls and 32 boys, with a mean age of 6.73 ± 2.93 years (range: 14 months to 13 years). Overall, 20 patients had left arm injuries and 22 patients had right arm injuries. Patient demographics, pattern of injury, and details of the surgical treatment were obtained from the patient notes. The causes of injury included: motor vehicle incident (2 patients), fall from height (32 patients), fall from bed (4 patients), fall from chair (1 patient), and fall from height of over 1 meter (3 patients). No patients experienced associated vascular injuries. There was 1 case of an open fracture (Gustilo and Anderson grade I). Four patients had a neurological deficit, involving the radial nerve and median nerve. All fresh injuries were classified according to the system developed by Bado.

All patients received preoperative X-ray scans to allow classification and planning. We included all patients with a follow-up time of over 1 year. Neglected Monteggia fracture was defined as presentation to the pediatric orthopedic surgeons over 2 weeks after the time of injury. Those requiring a secondary procedure after an initial failed treatment in another hospital (other than the application of an external fixator) were classified as a second-stage or salvage procedure. All 42 patients were divided into 2 groups according to their presentation time: 25 fractures were defined as fresh, with a presentation time of less than 2 weeks after injury, and the other 17 fractures were defined as neglected fractures, with a presentation time of over 2 weeks after injury. All clinical evaluations were undertaken by the same examiner, who remained blinded to the classification of injury in all patients. The function of the elbow was assessed using the Mayo Elbow Performance Score (MEPS, 90–100 = excellent, 75–89 = good, 60–74 = fair, <60 = poor) over 6 months post-operation. The study obtained ethical approval from the Review Board of Tongji Hospital ethical committee (number TJ-C20131211, 26/12/2013), and all patients gave written informed consent.

This was a retrospective case-control study. The fixation methods for ulna were divided into 6 types, and named from 0 to 5 according to the level of invasiveness (0, fixed by cast only; 1, fixed combined by polydioxanone suture (PDS) stich and cast; 2, fixed by combined triangular tension band and cast; 3, fixed by combined elastic stable intramedullary nail (ESIN) and cast; 4, fixed by combined plate and cast; 5, fixed by external fixator). The need to receive an open approach for correction of radial head dislocation was different between those 2 groups. Comparison of the different treatment methods was analyzed using nonparametric tests (the Wilcoxon signed rank test and the Chi-square test).

## 3 Results

### 3.1 Comparison of clinical characteristics

All of the patients had radial head dislocation and fracture of the ulna, which were identified by the initial X-ray examination (Table 1). Seventeen patients were included in the neglected group. However, all patients in the neglected group had received a previous reduction, cast, or surgery combined with a cast. The neglected group included 4 girls and 13 boys, with a mean age at the time of admittance of 6.94 ± 2.80 years (range: 2 years 1 month to 12 years 7 months). Seven patients had left arm injuries and 10 patients had right arm injuries. The average time between the date of accident and the date of treatment for radial head dislocation reduction was 4.82 ± 6.27 months (range: 1–25 months). Three patients presented with cubitus varus when admitted to our hospital. One patient developed joint pain, and 11 patients experienced limitation of extension and rotation movement. Two patients presented with symptoms of radial nerve injury. All injuries were classified according to the Bado system. There were 16 patients with anterior post-traumatic dislocation of the radial head, and 1 patient with lateral post-traumatic dislocation of the radial head. All patients received preoperative plain X-ray images to allow for classification and planning. Three patients had radial head re-dislocation within 1 year of follow up time and lacked full extension. All patients were evaluated according to the Mayo Elbow Performance Score, resulting in 10 excellent, 4 good, and 3 fair scores following treatment. No patients experienced associated vascular injuries. The average MEPS was 92.1 ± 9.3.

**Table 1:**
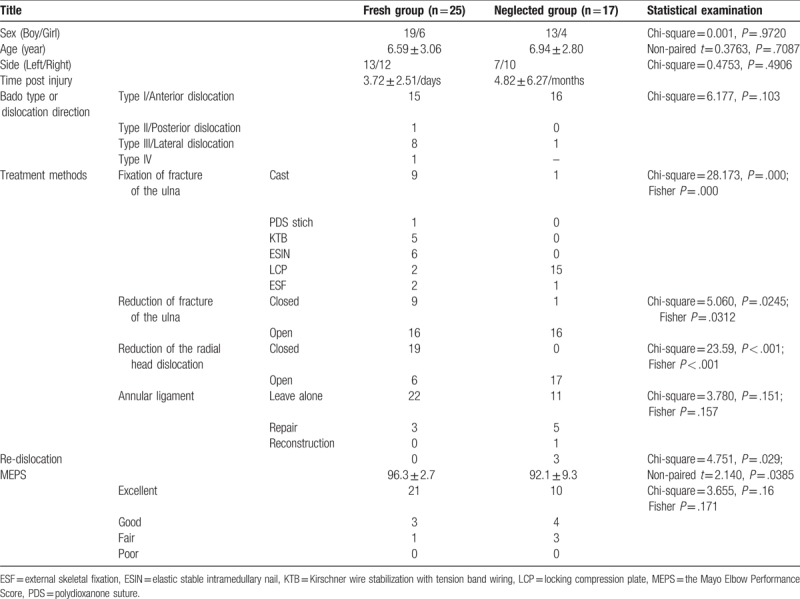
Details of the results in the 42 patients.

The other 25 patients (the fresh group) were diagnosed with radial head dislocation at the initial presentation, or no longer than 2 weeks after the time of injury. The fresh group included 6 girls and 19 boys, with a mean age at the time of admittance of 6.59 ± 3.06 years (range: 2 years 1 month to 12 years 7 months). Thirteen patients had left arm injuries and 12 patients had right arm injuries. The average time between the date of accident and the date of treatment for radial head dislocation reduction was 1.73 ± 1.70 days (range: 1 h to 5 days). One patient had suffered an open fracture. Two patients presented with symptoms of radial nerve and median nerve injury. All injuries were classified according to the Bado system: 15 patients were classified as Bado type I, 1 patient was classified as Bado type II, 8 patients were classified as Bado type III, and 1 patient was classified as Bado type IV. All patients received preoperative plain X-ray images to allow for classification and planning. No patients had radial head re-dislocation within 1 year of follow up time. All patients were evaluated according to the Mayo Elbow Performance Score, resulting in 21 excellent, 3 good, and 1 fair score following treatment. No patients experienced associated vascular injuries. The average MEPS was 96.3 ± 2.7.

All 42 patients were divided into 2 groups according to the interval between injury and reduction of radial head. The general clinical characteristics are listed in Table 1. Paired *t* tests suggested that there was no statistical significance in the age of the patients between the neglected group and the fresh group (*t* = 0.376, *P* = .709). Furthermore, Chi-square tests suggested that there were no significant differences in the sex or the side of affected arm between the neglected group and the fresh group (Pearson Chi-square = 0.001, *P* = .972; Pearson Chi-square = 0.475, *P* = .490, respectively). Thus, the two groups had similar clinical characteristics.

### 3.2 Case presentation 1: fresh Monteggia fracture managed with manipulation

**Figure 1:**
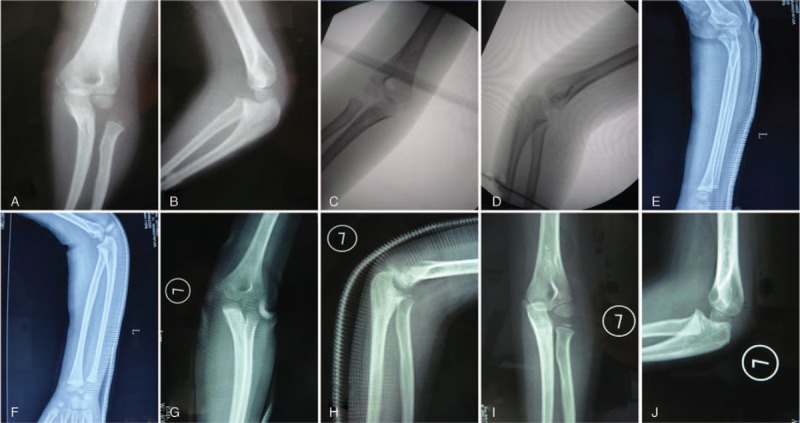
A 5-year-old girl presented 3 days after injury of her left elbow with initial type III Bado injury (A and B). She was treated with closed reduction of the ulna fracture and radial head, and application of a cast to immobilize the forearm (C–F). The cast was removed 6 weeks after the manipulation, and the girl was encouraged to begin movement of the elbow and gradually return to normal activities (G and H). X-ray images obtained 12 months after manipulation (I and J) showed maintenance of the reduction.

A 5-year-old Chinese girl presented with left elbow pain and elbow swelling lasting 3 days. She was diagnosed with an acute injury and was sent to Tongji Hospital, where she received an X-ray which revealed a proximal fracture of the ulna with an associated radial head dislocation (Fig. 1A and B). She was diagnosed with acute Monteggia fracture. She received manipulation and closed reduction and was treated with a cast (Fig. 1C– H). She recovered without any complications (Fig. 1I–J).

### 3.3 Case presentation 2: fresh Monteggia fracture managed with ESIN

**Figure 2:**
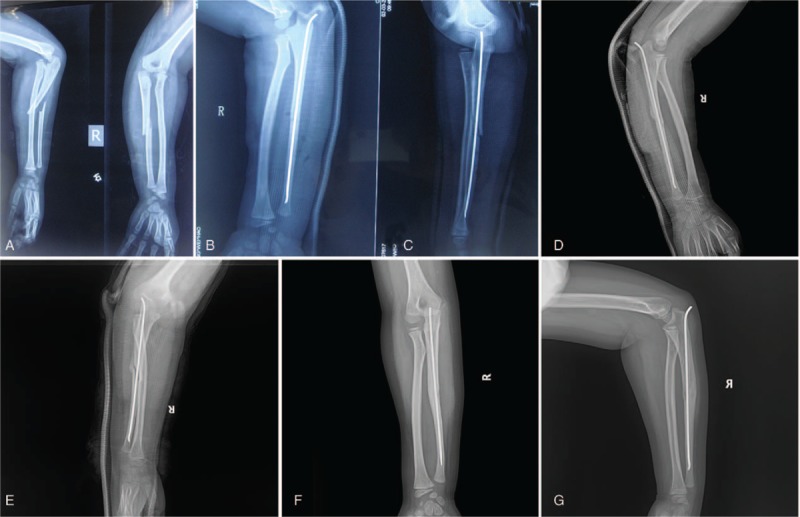
A 7-year-old boy was admitted 1 day after injury to the right elbow. The radiograph on presentation showed dislocation of the radial head (A and B). The elbow had loss of flexion due to elbow pain. We performed closed reduction of the radial head, closed reduction of the fracture of the ulna and insertion of an elastic stable intramedullary nail (B and C). Five weeks after the repair, the radiographs showed abundant bone callus at the ulna fracture, and the radial head remained reduced (D and E). The elastic stable intramedullary nail was removed and half a year after surgery, lateral and anteroposterior view of elbow illustrated good relationship between radial head and capitellum (F and G).

A 7-year-old Chinese boy presented with right elbow pain and elbow swelling lasting 1 day. He was diagnosed with an acute injury and was sent to Tongji Hospital, where he received an X-ray which revealed an oblique ulna fracture with an associated radial head dislocation (Fig. 2A). He was diagnosed with acute Monteggia fracture. He received manipulation and closed reduction, and was treated with an ESIN combined with a cast (B–E). This patient had an uneventful recovery (F– G).

### 3.4 Case presentation 3: neglected Monteggia fracture managed with LCP

**Figure 3:**
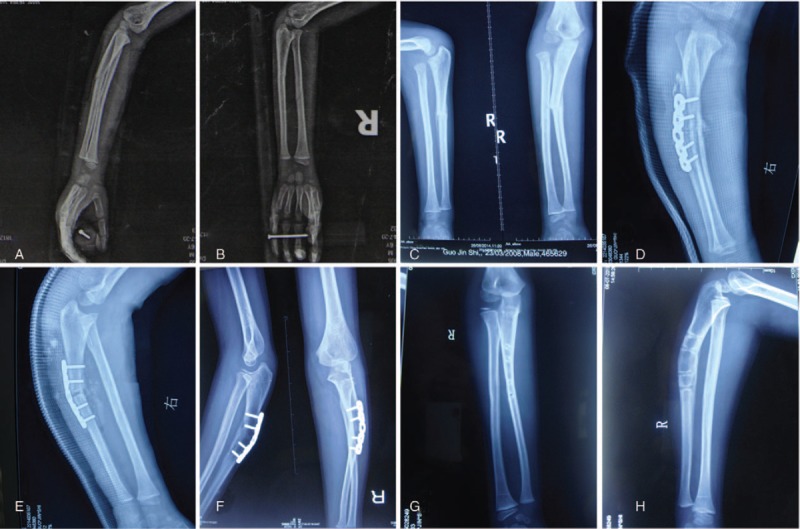
A 6-year and 5-month-old boy was admitted 2 months after injury to the right forearm. The radiograph on presentation showed complete dislocation of the radial head (A and B). The elbow had about 15-degree loss of flexion and the ulna fracture had united (C). We performed open reduction, osteotomy of the ulna at the site of CORA angle point and inserted a locking compression plate to stabilize the osteotomy of the ulna (D and E). Ten months after the repair, the radiographs showed complete bone union at the ulna osteotomy, and the radial head remained reduced (F). The plate was removed and 10 months after surgery, lateral and anteroposterior view of elbow illustrated good relationship between radial head and capitellum (G and H).

A 6-year-old Chinese boy presented with limited forearm rotation and elbow flexion. He had experienced an acute injury 2 months ago, and was sent to Tongji Hospital, where he received an X-ray which revealed a consolidated fracture of the ulna with an associated radial head dislocation (A and B). He was diagnosed with neglected Monteggia fracture (Fig. 3C). He received an open reduction of the radial head and osteotomy of the ulna and was treated with a LCP combined with a cast (Fig. 3D and E). This patient had an uneventful recovery (Fig. 3F–H).

### 3.5 Comparison of treatment methods for Monteggia fracture

After reviewing the treatment methods, all patients received closed or open reduction combined with postoperative immobilization with different types of stabilization, such as casts, suture fixation, Kirschner wire stabilization with tension band wiring (KTB), ESIN, LCP, and external skeletal fixation (ESF) (Table 2).

**Table 2:**
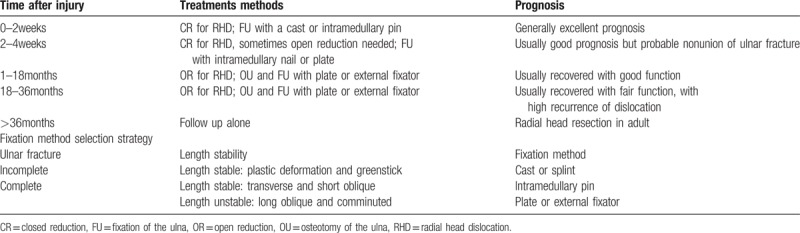
Treatment strategy upon the injury time.

**Figure 4:**
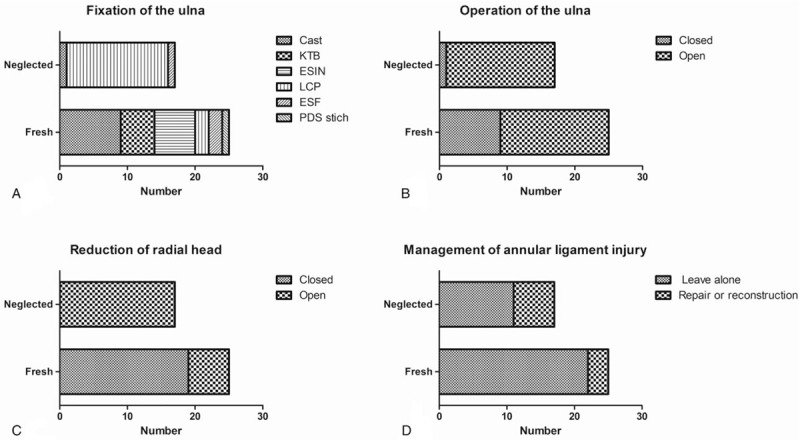
Treatment methods selected for fresh and neglected group. The distribution of fixation methods for stabilization of the ulna showed more frequent application of LCP in neglected group (A). Operative treatment was applied more frequently in the neglected group when compared to the fresh group (B). A higher rate of open radiocapitellar joint reduction was noted in the neglected group (C). Both groups showed a small number of annual ligament repair or reconstruction (D).

The distribution of postoperative immobilization methods was significantly different between the two groups (Chi-square = 28.173, *P* < .001). LCP was the most common choice for postoperative immobilization in patients of the neglected group (88.2%, Fig. 4A). Cast, ESIN, and KTB were the most common methods of fracture stabilization for patients in the fresh group (80%, Fig. 4A). The rate of closed reduction of the fracture of the ulna was also significantly different between the 2 groups (Chi-square = 5.060, *P* = .025). The rate of closed reduction of the fracture of the ulna was higher in the fresh group than in the neglected group (36% vs 5.9%, respectively, Chi-square = 5.060, *P* = .025; Fig. 4B).

The rate of closed reduction of the dislocated radial head was compared by the Chi-square test, which revealed a significant difference between the 2 groups (Chi-square = 23.59, *P* < .001). The closed reduction rate of the dislocated radial head in the fresh group was higher than that of the neglected group (76% vs 0%, respectively, Chi-square = 23.59, *P* < .001; Fig. 4C). The rate of annular ligament interference did not differ significantly between the 2 groups (Chi-square = 3.261, *P* = .071). The rate of reconstruction or repair of the annular ligament was higher in the neglected group than in the fresh group (35.3% vs 12%, respectively, Chi-square = 3.261, *P* = .071; Fig. 4D).

Furthermore, the prognosis was evaluated according to the MEPS scores and the rate of radial head dislocation recurrence. MEPS was higher in the fresh group compared to the neglected group (96.3 ± 2.7 vs 92.1 ± 9.3, *t* = 2.140, *P* = .039). The patients in the fresh group recovered with better elbow function (evaluated by MEPS) compared to the patients in neglected group (96% vs 82.4%, Chi-square = 2.054, *P* = .152). Radial head dislocation recurrence was another indicator of poor prognosis. Radial head dislocation reappeared in 3 patients of the neglected Monteggia fracture group, but in none of the fresh group (17.6% vs 0%, Chi-square = 4.751, *P* = .029).

### 3.6 Comparison of treatment methods for Monteggia fracture with anterior dislocation of the radial head

Anterior dislocation of the radial head is the most common type of Monteggia fracture in both the fresh and the neglected patients. We included all patients diagnosed with anterior dislocation of radial head (Table 1). All 31 patients were divided into 2 groups according to the interval between injury and reduction of radial head. The general clinical characteristics are listed in Table 1. We noted no statistically significant difference in the age of patients between the neglected group and the fresh group (*t* = 0.172, *P* = .865). There are no statistically significant differences in the sex and the side of the affected arm between the neglected group and the fresh group (Pearson Chi-square = 0.278, *P* = .598; Pearson Chi-square = 0.267, *P* = .605, respectively).

All patients received closed or open reduction combined with postoperative immobilization with different types of fixation, such as cast, stitches, KTB, ESIN, LCP and external skeletal fixation (ESF) (Table 2). The rate of closed reduction of the dislocated radial head was significantly different between the 2 groups (Chi-square = 23.88, *P* < .001). The rate of closed reduction of the dislocated radial head in the fresh group was higher than that of the neglected group (86.7% vs 0%). The rate of non-interference of the annular ligament was also significantly different between the 2 groups (Chi-square = 8.48, *P* = .004,). The rate of reconstruction or repair of the annular ligament in the neglected group was higher than that of the fresh group (43.8% vs 0%). The rate of closed reduction of the ulna did not differ significantly between the 2 groups (Chi-square = 3.638, *P* = .057). The rate of closed reduction of fracture of the ulna in the fresh group was higher than that of the neglected group (33.3% vs 6.3%, Chi-square = 3.638, *P* = .057,). The distribution of postoperative immobilization methods was compared using the Chi-square test, and significant differences were observed between the 2 groups (Chi-square = 18.12, *P* = .001). LCP was the most common choice for postoperative immobilization in the neglected group (87.5%). Meanwhile, cast, ESIN, and KTB were the most common choices for patients in the fresh group (73.3%).

Furthermore, the prognosis was evaluated according to the MEPS score and the rate of radial head dislocation recurrence. The MEPS score of the fresh group was higher than that of the neglected group (97.2 ± 2.9 vs 92.6 ± 9.4; *t* = 1.81, *P* = .08). The patients in the fresh group recovered with better elbow function (evaluated by MEPS) compared to the patients of the neglected group (93.3% vs 68.8%, Chi-square = 1.006, *P* = .316,). Radial head dislocation recurrence was another indicator for poor prognosis. Radial head dislocation reappeared in 3 of the neglected Monteggia fracture patients but in no patient of the fresh group (18.75% vs 0%, Chi-square = 3.114, *P* = .078).

## 4 Discussion

Monteggia fractures, that is, fractures of the ulna with dislocation of the radial head, are uncommon. This study included a moderate number of pediatric patients with clearly defined Monteggia fractures in a single clinic center.

Mid and long-term dislocation of the radial head increases the probability of developing radial head dysplasia. Union of the fracture of the ulna represents no problem in acute Monteggia injuries. However, by the time neglected Monteggia lesions were reviewed, the fracture of the ulna had already united. It is more difficult to diagnose neglected Monteggia fracture in patients with plastic deformation of the ulna or if the ulna fracture has already united.

Many authors recommend closed reduction and cast immobilization for acute Monteggia fracture dislocations. For patients with fresh Monteggia fractures, closed reduction combined with a cast is 1 of the most common treatment options, and usually results in an uneventful recovery.^[5,6]^ But failure, or loss of reduction and missed dislocation, may require a more invasive operation. Excellent outcomes are usually anticipated following appropriate treatment of acute Monteggia injuries. However, the complex and often unpredictable results of surgical reconstruction for chronic or missed Monteggia lesions further highlight the importance of proper initial recognition and reduction.^[6–10]^ Many studies have investigated the various options for treatment of acute Monteggia fractures, with recommendations ranging from closed reduction and casting alone, to operative fixation of acute injuries according to the fracture pattern of the ulna.^[6,11,12]^

In efforts to optimize management of acute Monteggia fracture dislocations, Ring and Waters proposed a treatment strategy based upon the pattern of fracture of the ulna.^[12,13]^ Their guiding principle in the treatment of these injuries was restoration and maintenance of alignment of the ulna. Although incomplete fractures of the ulna (eg, plastic deformation and greenstick fractures) may be successfully managed with closed reduction and casting, surgical reduction and stabilization of complete fractures was recommended. For "length stable” fractures of the ulna (eg, transverse or short oblique), intramedullary pin fixation was recommended. For "length unstable” fractures of the ulna (eg, long oblique or comminuted), open reduction and plate fixation was recommended (Table 2). Despite the existing debate surrounding the most effective treatment strategy, the report by Ring and Waters is a valuable guideline regarding the systemic advices for treatment methods of fresh Monteggia fracture.

According to our retrospective study, closed reduction combined with a cast, ESIN and the KTB after manipulation were the most common choices for patients in the fresh group. Nearly all patients in the neglected group required more invasive operations, such as osteotomy of the ulna with LCP internal fixation combined with an open reduction of the dislocated radial head. Our study shows that the rates of patients that needed open reduction or complex immobilization methods was higher in the neglected group compared to those of the fresh group. Therefore, only by early discovery of Monteggia fractures, we can rely upon minimally invasive methods. The fixation of forearm fractures is aimed to benefit the correct alignment of the radiocapitellar joint to avoid recurrent dislocation, which is also a benefit of cast immobilization. Closed reduction was the most commonly applied treatment option for the fresh group. However, open reduction was also applied in the treatment of fresh Monteggia fractures, in the case of failure of closed radiocapitellar joint reduction.

Radial head dislocation can be classified as neglected if the fracture of the ulna has united or is uniting by the time of diagnosis. Many studies use a cut-off of 4 weeks before considering a Monteggia lesion to be ‘neglected’.^[7,14]^ This is because the radial head is considered to be irreducible using closed means by this time. However, we used a cut-off of 2 weeks, as this is enough time for young children to develop strong bone callus, preventing successful reduction. Therefore, a more detailed plan is required for pediatric patients with neglected Monteggia fractures before operation.

Union of the fracture of the ulna is a problem that requires extra attention during this time period. Osteotomy of the ulna represents the widely applied treatment for radial head dislocation after a missed Monteggia fracture. The concept of osteotomy of the ulna is: the osteotomy tightens the interosseous membrane sufficiently to keep the radial head in the correct anatomical position. To preserve all of the interosseous membrane and to use its tension to pull the radial head posteriorly, the osteotomy should be more proximal than the junction of the proximal quarter and the distal three-quarters of the ulna. Regarding the surgical procedures for the reconstruction of the annular ligament, several methods have been proposed: using a free palmaris longus tendon, pedicled forearm fascia, pedicled fascia of the triceps, and suture repair of the remnants of the annular ligament. After reconstruction of the annular ligament, a radial neck notch, signaling constriction of the radial neck by the reconstructed ligament, has often been observed on postoperative radiographs. Therefore, the treatment of neglected Monteggia fractures is still a great challenge for orthopedic surgeons.

In this retrospective comparison study, the fresh group recovered better than the neglected group, with higher MEPS scores and lower rates of recurrent dislocation. The prognosis was improved when the fracture was diagnosed within the first 2 weeks following injury. If the fracture is not diagnosed on initial presentation, and remains undiscovered for over 2 weeks, it is difficult to achieve an excellent or good MEPS score after treatment.

Delayed diagnosis and treatment of radial head dislocation will cause the classic complications in the treatment of Monteggia fracture. If left untreated, the dislocated radial head becomes symptomatic, causing elbow pain, decreased elbow motion, increasing valgus deformity, and neurologic problems.^[15]^ Osteotomy of the ulna will be required to repair Monteggia injuries, thus bringing huge trauma to those patients.^[16]^ However, early diagnosis and adequate treatment of Monteggia fractures result in excellent outcomes, and allow for conservative management in most cases, without complex surgical procedures involving osteotomies of the ulna, or repair or reconstruction of the annular ligament. On the other hand, some pathologic changes may develop to prevent reduction of the radial head when the diagnosis is delayed.^[17]^ Therefore, the appropriate treatment of Monteggia injury should be applied as soon as possible, and patients should be transferred to specialized pediatric orthopedic surgeons to avoid a delayed diagnosis.

However, this study has several limitations. First, this was a retrospective study without randomization. Our sample size was small because of the low incidence of Monteggia fractures in pediatric patients. This will decrease the power of statistical analysis. Second, subgroup analysis was performed according to diagnosis of the radial head dislocation. Thus, objective comparison was not entirely possible for both groups. Furthermore, most of the patients required reduction and/or reconstruction of the annular ligament injury. Therefore, there is bias in personal experiences of treatment methods. Also, there is dispute regarding the surgical treatment of the annular ligament. Thus, further prospective studies are required to explore the best options for management of the annular ligament in neglected Monteggia fractures.

## 5 Conclusion

A good outcome after a Monteggia fracture in pediatric patients requires early diagnosis of radial head dislocation and prompt, stable, anatomical reduction of the fracture of the ulna. In our experience, selective operative fixation of unstable fractures provides reliable reduction and causes fewer complications.

## Author contributions

**Conceptualization:** Jin Peng He, Yun Hao, and Jing Fan Shao.

**Data curation:** Jin Peng He and Yun Hao.

**Formal analysis:** Jin Peng He and Yun Hao.

**Funding acquisition:** Jin Peng He.

**Investigation:** Jin Peng He and Yun Hao.

**Methodology:** Jin Peng He and Yun Hao.

**Project administration:** Jin Peng He.

**Resources:** Jin Peng He and Yun Hao.

**Software:** Jin Peng He and Yun Hao.

**Supervision:** Jin Peng He and Jing Fan Shao.

**Validation:** Jin Peng He.

**Visualization:** Jin Peng He and Jing Fan Shao.

**Writing – original draft:** Jin Peng He.

**Writing – review & editing:** Jin Peng He.

Jin Peng He orcid: 0000-0001-7089-6955.
